# Bibliometric Analysis of the Worldwide Scientific Production on Periapical Lesions in Veterinary Medicine: Visualization, Emerging Patterns, and Thematic Evolution

**DOI:** 10.1155/vmi/4483647

**Published:** 2026-05-14

**Authors:** Gabriel Barriga-Yauri, Daniel Alvitez-Temoche, Lucia Quispe-Tasayco, Julia Medina, Fran Espinoza-Carhuancho, Josmel Pacheco-Mendoza, Frank Mayta-Tovalino

**Affiliations:** ^1^ Academic Department, Research, Innovation and Entrepreneurship Unit, Faculty of Dentistry, Universidad Nacional Federico Villarreal, Lima, Peru, unfv.edu.pe; ^2^ Academic Department, Bibliometrics, Evidence Assessment and Systematic Reviews (BEERS) Group, Human Medicine Career, Universidad Cientifica del Sur, Lima, Peru, ucsur.edu.pe; ^3^ Academic Department, EVIDENTIA Research Group, Universidad Nacional Mayor de San Marcos, Lima, Peru, unmsm.edu.pe; ^4^ Academic Department, Vice-Rectorate for Research, Universidad San Ignacio de Loyola, Lima, Peru, usil.edu.pe

**Keywords:** bibliometric mapping, periapical lesions, veterinary medicine

## Abstract

To conduct a bibliometric mapping of the global scientific production of periapical lesions in veterinary medicine by evaluating collaborative networks, emerging patterns, and spatiotemporal dynamics. Documents published between 1986 and 2024 that addressed topics related to periapical lesions in the veterinary field were included. Articles, reviews, book chapters, and books containing specific key terms in their titles or abstracts, such as “periapical abscess,” “periapical infection,” and “periapical pathology,” were selected. Documents unrelated to the veterinary field, duplicates, and those that did not provide relevant information were excluded. The search was conducted in Scopus on December 28, 2024, using a specific search formula. The data selection and extraction process was conducted using RStudio and the Bibliometrix package, and VOSviewer was used for the co‐occurrence analysis of keywords. Lotka’s law, Bradford’s law, and thematic evolution were applied for data analysis. The study covered the period from 1986 to 2024 and analyzed 95 documents from 37 sources with an annual growth rate of 2.93%. The average age of the documents was 12.2 years, with an average of 13.96 citations per document and 2345 references. A total of 280 authors participated, with only 15 single‐author documents and an average of 3.83 co‐authors per document. International collaborations accounted for 11.58% of the total. The most productive journals were the “*Journal of Comparative Pathology*” and the “*Journal of Veterinary Dentistry*.” Research in veterinary dentistry showed a significant increase between 2012 and 2024, highlighting topics such as dental pathology and periapical infection. The keyword co‐occurrence analysis revealed four main clusters: “Dental pathology,” “Dog,” “Horse,” and “Root canal treatment.” The field of dental pathology holds significant promise for continued research, especially in the creation of innovative treatments and therapeutic applications for animal dental issues. Global collaborations and the identification of thematic clusters offer a robust framework for steering future research toward emerging and impactful areas.

## 1. Introduction

Periapical lesions are osteolytic conditions that develop in the apical region of the tooth root [1]. These lesions can significantly impact an individual’s quality of life, making it crucial to understand their formation process and identify more effective and sustainable treatments [2]. They are commonly defined as infections that primarily affect the tooth root and surrounding alveolar bone, typically caused by gram‐negative bacteria entering the endodontic system, leading to tissue degradation in the apical region of the tooth [1, 3].

These lesions can also arise from untreated dental caries, wear, and dental trauma, potentially leading to pulp necrosis [4]. Periapical granulomas and radicular cysts, which are the inflammatory origins of periapical lesions, are associated with apical periodontitis and are distinguished by the absence or presence of an epithelial lining [5].

Dental diseases are prevalent across various species and can lead to significant health issues, particularly in animals. Identifying and analyzing periapical lesions are crucial for providing appropriate dental treatment [6]. Several studies in veterinary medicine highlight experiments on rats and dogs, demonstrating the use of mineral trioxide aggregate (MTA) and epigallocatechin gallate (EGCG)–based paste [7, 8]. These substances are influential in healing periapical lesions in animals, promoting cellular development, regulating alveolar bone regeneration in the periapical area, creating antibacterial environments, and improving the histopathological repair of these lesions [9, 10].

Bibliometrics is a discipline that employs quantitative and statistical methods to study the production and dissemination of scientific literature, analyzing data such as citations, co‐authorship, and publication zones [11]. It offers various benefits, including evaluating research impact, conducting detailed analyses of scientific productivity, and tracking the evolution and influence of studies over time. In addition, it facilitates the identification of trends, the recognition of emerging areas, and the promotion of collaborations, contributing to strategic planning and efficient resource allocation in research institutions [12].

Bibliometric visualization is applied to the study of periapical lesions in veterinary medicine. These maps delineate the body of knowledge residing in the meta information, enabling us to grasp how periodontitis, along with the periapical lesions that arise, is constituted. Thus, we can germinate future work, optimize resources, and foster integration of each sector in veterinary dentistry. Thus, this study aimed to conduct a bibliometric mapping of the global scientific production of periapical lesions in veterinary medicine by evaluating visualization, emerging patterns, and thematic evolution.

## 2. Methods

### 2.1. Study Design

This study utilized a descriptive design combined with a bibliometric approach to examine the scientific literature on periapical lesions in animals. The Reporting and Measurement of Items for Bibliometric or Scientometric Studies (RAMIBS) in Health Sciences guideline, a customized tool for drafting bibliometric articles, was used for this study [13].

### 2.2. Inclusion and Exclusion Criteria

This study included documents published between 1986 and 2024 that focused on periapical lesions in the veterinary field. Selected materials comprised articles, reviews, book chapters, and books with specific key terms in their titles or abstracts, such as “periapical abscess,” “periapical infection,” and “periapical pathology.” Excluded were documents unrelated to the veterinary field, those not meeting the established search criteria, duplicate publications, and those lacking relevant information on the study topic.

### 2.3. Search Strategy

The search was conducted using the Scopus database on December 28, 2024. The following search formula was used: TITLE‐ABS (“Alveolar Abscess Apical” OR “Abscess Apical Alveolar” OR “Abscesses Apical Alveolar” OR “Alveolar Abscesses Apical” OR “Apical Alveolar Abscess” OR “Apical Alveolar Abscesses” OR “Dentoalveolar Abscess Apical” OR “Abscess Apical Dentoalveolar” OR “Abscesses Apical Dentoalveolar” OR “Apical Dentoalveolar Abscess” OR “Apical Dentoalveolar Abscesses” OR “Dentoalveolar Abscesses Apical” OR “Abscess Periapical” OR “Abscesses Periapical” OR “Periapical Abscesses” OR “Periapical Periodontitis Suppurative” OR “Periapical Periodontitis Suppurative” OR “Periodontitides Suppurative Periapical” OR “Periapical Infection” OR “Periapical Lesion” OR “Periapical Granuloma” OR “Periapical Cyst” OR “Periapical Pathology” OR “Periapical Disease” OR “Periapical Inflammation” OR “Periapical Abscess” OR “Periapical Abscesses” OR “Periapical Periodontitis” OR “Periapical Periodontitides” OR “Periapical Suppuration” OR “Periapical Suppurations” OR “Periapical Suppurative Lesion” OR “Periapical Suppurative Lesions” OR “Periapical Suppurative Infection” OR “Periapical Suppurative Infections”) AND SUBJAREA (vete).

### 2.4. Data Source

Scopus was selected as the only data source for this study because it is one of the largest multidisciplinary databases and is regarded for its comprehensive coverage of health and veterinary sciences literature, providing comparatively better indexing for journals, books, and conference proceedings when compared to rival databases. The search tools and the standard controlled vocabulary also help to make bibliometric investigations replicable and transparent. Finally, it provides good citation information and reliable author affiliation details.

### 2.5. Procedures

The data selection and extraction process was carried out using R Studio and the Bibliometrix package. Initially, documents retrieved from Scopus were imported, and the data were cleaned to remove duplicates and irrelevant entries. Subsequently, bibliometric metrics such as the number of publications, citations, and international collaborations were analyzed. For the co‐occurrence analysis of keywords, VOSviewer was employed, enabling the visualization of relationships between terms and the identification of thematic clusters.

### 2.6. Data Analysis

The bibliometric analysis plan involved evaluating various metrics, including the annual growth rate, the average age of documents, the average number of citations per document, and the total number of references. R Studio and the Bibliometrix package were utilized for data selection and extraction, as well as for analyzing bibliometric metrics. In addition, VOSviewer was used to conduct the co‐occurrence analysis of keywords, allowing for the identification of main thematic clusters and the visualization of relationships between terms. Lotka’s law was applied to analyze author productivity, Bradford’s law to identify the most productive journals, and thematic evolution to observe research trends over time. These tools and metrics provide a comprehensive overview of scientific production and collaborations in research on periapical lesions in veterinary medicine.

## 3. Results

### 3.1. Main Information

The period covered by the study is 1986–2024, inclusive of the boundary years. Num. doc: 95 and sources: 37. The annual growth rate is 2.93% (should be treated with circumspection). Three periods were apparent: (i) A low‐output period from 1986 to 2000; (ii) an increase in output from 2001 to 2011; and (iii) an increase in output from 2012 to 2024, when there was an explosion in papers pertaining to veterinary dentistry. The most productive year period was the last 10 years, when this discipline began to grow legs! Overall, the average age of documents was 12.2. The average number of citations per document is 13.96. The total number of references was 2345. From the authors′ keywords, there were 245 unique terms. There were 280 authors, 15 single‐author docs, 3.83 co‐authors/document, and 11.58% international collaborations. Types of documents: 87 articles, 1 book, 3 book chapters, and 4 reviews (Table 1).

**TABLE 1:** Main information.

Description	Results
Timespan	1986:2024
Sources	37
Documents	95
Annual growth (%)	2.93
Document average age	12.2
Average citations per doc	13.96
References	2345
Author’s keywords	245
Authors	280
Authors of single‐authored docs	15
Single‐authored docs	17
Co‐Authors per doc	3.83
International co‐authorships (%)	11.58
Article	87
Book	1
Book chapter	3
Review	4

### 3.2. Lotka’s Law

**FIGURE 1:**
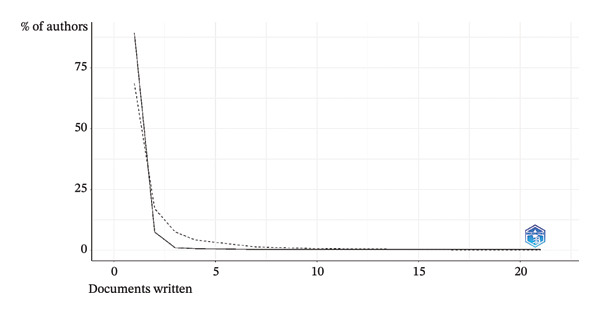
Lotka’s law.

The analysis according to Lotka’s law revealed a clear trend in scientific production. A total of 250 authors (89.3% of the total) wrote one document. In contrast, 21 authors produced two documents, representing 7.5% of the authors. In addition, 3 authors wrote three documents, representing 1.1% of the total. Finally, a small group of authors, specifically 1 author, contributed seven documents, another 10 documents, another 17 documents, and another 21 documents, each representing 0.4% of the authors (Figure 1).

### 3.3. Bradford’s Law

**FIGURE 2:**
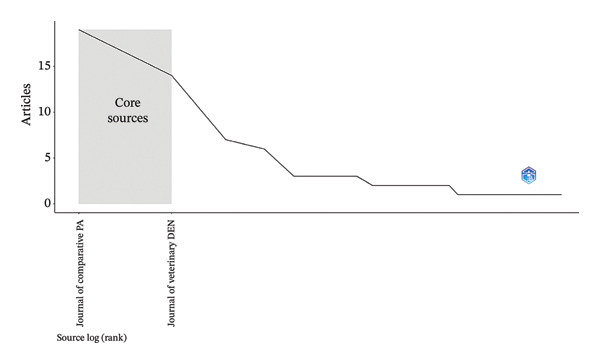
Bradford’s law.

According to Bradford’s law, the most productive journals in the research on periapical lesions in veterinary medicine were the “*Journal of Comparative Pathology*” and the “*Journal of Veterinary Dentistry.”* Zone 2 included journals such as “*Equine Veterinary Education*” and the “*Journal of the American Veterinary Medical Association.”* In Zone 3, journals such as “*Journal of Small Animal Practice*” and “*Veterinary Clinics of North America-Exotic Animal Practice*” were found. This distribution highlights the main sources of publications in this field (Figure 2).

### 3.4. Thematic Evolution

**FIGURE 3:**
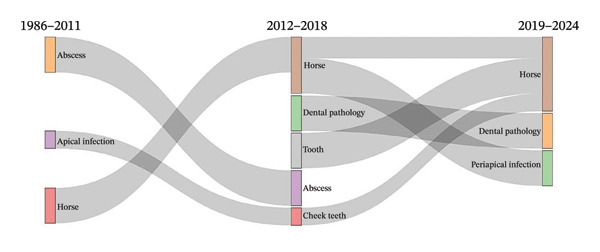
Thematic evolution.

The thematic evolution analysis showed that studies on abscesses and horses were constant throughout the whole period, spanning 1986–2024, and form the oldest and stable research line. Topics studying apical infections and molar teeth increased considerably between 2012 and 2024, coinciding with the growth of veterinary dentistry. Nodes studying the pathology of teeth and temporomandibular joints appeared repeatedly in recent years, indicating growing clinical relevance. Periapical infection, which appeared randomly initially, became central in the last 10 years, being one of the more interconnected nodes within the network. By supplementing each node with its frequency, time of appearance, and relative weight, we obtain a clearer view of the evolution and temporal patterns of research themes (Figure 3), departing from the consolidated areas of abscesses and horses into more recent proliferations such as dogs, root canal treatment, and periapical infection. Our findings illustrate the multidimensionality of veterinary research on lesions of periapex and their progression in time (Figure 3).

### 3.5. Country Collaboration Map

**FIGURE 4:**
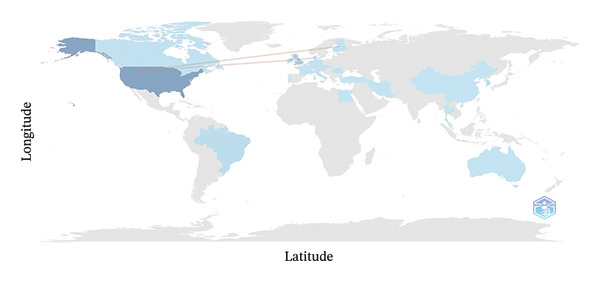
Country collaboration map.

The analysis of the collaboration map among countries revealed various international interactions in research on periapical lesions in veterinary medicine. Notable collaborations included those between the United States and several countries, including Austria, Canada, China, Denmark, Finland, Thailand, and the United Kingdom. The United Kingdom also showed significant collaborations with Canada, Denmark, and Ireland. Other countries, such as Denmark, Germany, and Slovenia, have also participated in international collaborations with Canada, Austria, and the Czech Republic. These data underscore the global and collaborative nature of research in this field (Figure 4).

### 3.6. Co‐Occurrence by Keyword

**FIGURE 5:**
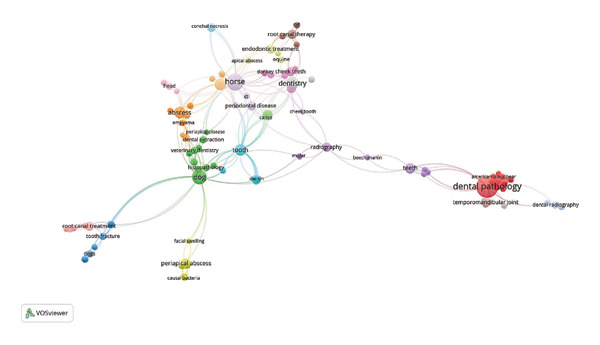
Co‐occurrence by keyword.

The keyword co‐occurrence analysis revealed four main clusters of periapical lesions in veterinary medicine research. The red cluster, named “Dental pathology,” was the largest and showed a strong interrelation with other clusters, indicating that dental pathology is a central theme in this field. The green cluster, “Dog,” highlighted specific research on dogs, while the purple cluster, “Horse,” emphasized the importance of studies on horses. The pink cluster, “Root canal treatment,” focused on root canal treatments and showed significant connections with other clusters. These interrelations suggest that research on periapical lesions in veterinary medicine is multidimensional and encompasses various species and treatments, with dental pathology as the central axis (Figure 5).

## 4. Discussion

Currently, it is crucial to investigate animals as models to analyze various periapical lesions, such as apical periodontitis, which affects half of the adult population and can manifest as acute or chronic depending on the infection [14, 15]. However, human physiology is not fully reflected in these models because these diseases largely depend on the host organism [16, 17].

Despite this, studies on periapical lesions in veterinary medicine have gained relevance in recent years because of experiments conducted on various species to address different associated pathologies. This information is reflected in our study, which recorded 95 documents from 1986 to 2024, with an annual growth rate of 2.93%. Therefore, it is of utmost importance to conduct studies quantifying the information obtained to date.

A bibliometric study conducted by Ferraz et al. analyzed the 100 most cited publications on AP and systemic health, highlighting the collaboration of countries such as Brazil with 25 articles and 1074 citations, the United States with 21 articles and 934 citations, and Spain with 14 articles and 895 citations [18]. These findings align with our study, in which countries from the Americas and Europe contributed the most articles to the research. Europe’s participation in various investigations related to these diseases coincides with and corroborates other studies that position it as the leading continent in endodontic research due to its high contribution [19–21].

In addition, another bibliometric study by Yeung AWK aimed to reveal which radiolucent lesions are most frequently mentioned, concluding that some of the author’s keywords did not coexist, while those that did were apical periodontitis and ameloblastoma with greater relevance. In contrast, our study keywords were dental pathology, dog, horse, and root canal treatment [22].

Furthermore, the study highlighted the most recurrent journals, including *Oral Surgery, Oral Medicine, Oral Pathology, Oral Radiology, Journal of Endodontics, Dentomaxillofacial, International Endodontic Journal,* and *Journal of Oral and Maxillofacial Surgery*. Unlike our analysis, which employed Bradford’s law, the most productive journals *were Journal of Comparative Pathology, Journal of Veterinary Dentistry, Equine Veterinary Education,* and *Journal of the American Veterinary Medical Association* [22].

In addition, Grillo et al. performed a global scientific mapping study focused on odontogenic infections. In total, the study identified 1661 publications predominantly written in English. These articles received 22,041 citations, with an average of 13.27 citations per publication. Furthermore, the study revealed an H‐index of 63 in this field of research, indicating a significant contribution to the area of odontogenic infections [23].

The considerable rise in publications is due to the intersection of several factors. On the one hand, diagnostic imaging and molecular biology advancements have led to improved detection and characterization of periapical lesions in animals, and even comparative studies in human dentistry. Simultaneously, the recent focus on animal health and welfare as part of the One Health concept has increased visibility of veterinary dentistry related to the understanding of oral diseases and increased interdisciplinary interest and funding. Even though international collaboration accounted for only 11.58% of the total output, collaborations among the United States, the United Kingdom, Canada, and a handful of European countries were responsible for many of the cited works and provided pathways to visibility and methods that were unique to the work originating from those countries. At the same time, regional research development, especially in Europe and North America, provided institutional support, access to journals, and research training programs that socialized veterinary dentistry into a distinct developing discipline. Overall, these dynamics lead to the conclusion that this increase in scientific production is not only increasing in volume but also the nature of the science is changing due to technological innovation, global health prioritization, and the establishment of collaborations with regional research systems [21–23].

Recent publications about periapical lesions in veterinary medicine show that many factors have come together to create the current research environment. The availability of advanced imaging techniques and the introduction of molecular biology to help identify and define these lesions have encouraged new developments in veterinary dentistry. In addition, within the context of One Health, the growing public and professional awareness regarding animal welfare has increased the need for researching these conditions; thus, international collaborations regarding research on periapical lesions are not widely utilized today. However, international collaboration has enabled the sharing of valuable ideas and methods, which have allowed for the emergence of veterinary dentistry as a distinct area of veterinary medicine. While regional initiatives have been important for building a foundation to support the development of a specialized field of veterinary dentistry, these initiatives have played a crucial role in providing an avenue for increased research, publishing activity, and training opportunities related to veterinary dentistry. In sum, these factors all explain the increase in scientific output related to the topic of periapical lesions in veterinary medicine, as well as influence the evolution of how researchers in different geographical areas approach the subject of periapical lesions and how they document their work through bibliometric analysis [23–26].

This study has several important limitations that should be acknowledged [24]. The most notable finding is the lack of specific bibliometric studies on periapical lesions in veterinary medicine. This gap in the literature underscores the significance of our research, as it lays the groundwork for future investigations [25] and may spark interest in this underexplored field [26]. Another key limitation was that the information search was confined to the Scopus database. Consequently, other relevant studies published in different databases, such as PubMed and Web of Science, were not included in our research. The exclusion of these sources may have restricted the scope and depth of the analysis, as well as the potential to obtain a more comprehensive view of the topic. Despite these limitations, this study sets a precedent, paving the way for new research that could address periapical lesions from various perspectives using a broader approach that includes multiple databases and methodologies.

We also emphasize that we have found, until now, no bibliometric studies focused on periapical lesions in veterinary literature. This is the reason why we compared our results to those conducted on fields such as human dentistry, even if those mentioned studies are not fully comparable; we believe this as reference to another field approximates more our data and highlights the particularities that veterinary studies have, such as the reliance on models, others species, the variables related to reported anatomy, and the recent formal emergence of this discipline.

## 5. Conclusion

Research in veterinary dentistry saw a notable increase between 2012 and 2024, focusing on topics such as dental pathology and periapical infection. International collaborations made up 11.58% of the total, highlighting the global scope of this research. The most productive journals were the “*Journal of Comparative Pathology*” and the “*Journal of Veterinary Dentistry*.” The keyword co‐occurrence analysis revealed four main clusters: “Dental pathology,” “Dog,” “Horse,” and “Root canal treatment,” indicating multidimensional research encompassing various species and treatments. These findings suggest that dental pathology remains a promising area for further research, particularly in the development of new treatments and therapeutic applications for dental problems in animals.

## Author Contributions

Gabriel Barriga‐Yauri, Frank Mayta‐Tovalino, and Fran Espinoza‐Carhuancho searched the database and analyzed the data. Daniel Alvitez‐Temoche, Lucia Quispe‐Tasayco, Julia Medina, Fran Espinoza‐Carhuancho, and Frank Mayta‐Tovalino wrote the main manuscript text and prepared the table. Fran Espinoza‐Carhuancho and Frank Mayta‐Tovalino analyzed the data. Frank Mayta‐Tovalino, Lucia Quispe‐Tasayco, Gabriel Barriga‐Yauri, Julia Medina, Fran Espinoza‐Carhuancho, and Josmel Pacheco‐Mendoza designed, critically reviewed, and modified the manuscript.

## Funding

No funding was received for this study.

## Disclosure

All authors have read and approved the final manuscript.

## Conflicts of Interest

The authors declare no conflicts of interest.

## Data Availability

The data that support the findings of this study are available from the corresponding author upon reasonable request.
